# Mutational Landscape in Resected Periampullary Adenocarcinoma: Relationship With Morphology and Clinical Outcome

**DOI:** 10.1200/PO.18.00323

**Published:** 2019-03-21

**Authors:** Sebastian Lundgren, Sofie Olsson Hau, Jacob Elebro, Margareta Heby, Emelie Karnevi, Björn Nodin, Jakob Eberhard, Karolina Holm, Johan Staaf, Göran B. Jönsson, Karin Jirström

**Affiliations:** ^1^ Lund University, Lund, Sweden

## Abstract

**PURPOSE:**

Periampullary adenocarcinomas encompass a heterogeneous group of tumors with dismal prognosis and limited treatment options. Emerging evidence shows that tumor morphology (ie, intestinal type [I-type] or pancreatobiliary type [PB-type]) is a more relevant prognostic factor than tumor origin. Knowledge is sparse, however, on whether key mutations differ according to morphology.

**MATERIALS AND METHODS:**

Next-generation sequencing was applied to assess the mutational status of 70 genes in 102 tumors from a retrospective cohort of 175 patients with resected periampullary adenocarcinoma. Brahma-related gene 1 protein expression was examined by immunohistochemistry on tissue microarrays with primary tumors from the original cohort.

**RESULTS:**

*APC* mutations were significantly more common in I-type than in PB-type tumors (27.5% *v* 0%; *P* < .001), as were *ERBB3* mutations (20.8% *v* 4.8%; *P* = .016), whereas *CDKN2A* mutations were more common in PB-type than in I-type tumors (19.4% *v* 2.5%; *P* = .013). *KRAS* mutation was an independent factor of poor prognosis in I-type tumors (hazard ratio, 3.73; 95% CI, 1.10 to 12.67). In PB-type tumors, *SMARCA4* mutation was an adverse prognostic factor in patients not receiving adjuvant chemotherapy, and there was a significant treatment interaction between expression of Brahma-related gene 1 protein, the protein encoded by *SMARCA4*, and adjuvant chemotherapy (*P*_interaction_ = .007).

**CONCLUSION:**

To our knowledge, this is the first description of the mutational landscape in the full spectrum of periampullary adenocarcinoma that demonstrates that the distribution and prognostic and predictive significance of commonly mutated genes differ by morphology. The results emphasize that morphology is an important factor to consider in the search for novel biomarkers and targeted personalized treatment of these patients. In addition, the findings support the concept that molecular profiling of these tumors could be of clinical benefit.

## INTRODUCTION

Periampullary adenocarcinoma is a collective term for tumors arising in the area surrounding the ampulla of Vater, including the head of the pancreas, the duodenum, and the common bile duct. A morphologic classification into intestinal type (I-type) and pancreatobiliary type (PB-type) has been shown to provide better prognostic information than anatomic origin, with the former having a more favorable clinical outcome.^1-3^ Although a plethora of mutations has been documented in pancreatic cancer,^4-7^ the mutational landscape of other periampullary cancers has been less studied. No targeted therapies have proven to be efficient, and adjuvant chemotherapy therefore remains standard of care after resection of these tumors. Hence, there is evident need for additional studies on the distribution and clinical significance of the mutational landscape in the full range of periampullary adenocarcinomas, including pancreatic cancer, to enable a better patient stratification and identify potential responders to targeted therapies. The aim of this study was therefore to map mutations in common cancer-associated genes in tumors from a clinically well-characterized retrospective cohort of patients with resected periampullary adenocarcinoma, with particular reference to morphology and clinical outcome.

## MATERIALS AND METHODS

### Study Cohort

The original study cohort is a retrospective consecutive series consisting of primary tumors from 175 incident cases of periampullary adenocarcinoma.^8,9^ The patients underwent pancreaticoduodenectomy at the University Hospitals of Lund and Malmö from January 1, 2001 to December 31, 2011. Follow-up started at the date of surgery and ended at death, or on March 31, 2017, whichever came first. The Swedish National Civil Register was used to obtain information on vital status. Data on adjuvant treatment were obtained from patient charts. All cases underwent thorough histopathological revaluation. The anatomic site of origin of the tumors was 14 duodenal, 70 ampullary, 45 distal bile duct, and 46 pancreatic, whereof 65 tumors were classified as intestinal type (I-type) and 110 as pancreatobiliary type (PB-type).

**Context**

- To what extent does morphology influence the mutational landscape in periampullary adenocarcinomas, and can we find novel prognostic and predictive biomarkers that differ by morphology, so as to better characterize this heterogeneous group of tumors in a clinical context?
- The results presented here demonstrate that the distribution and prognostic impact of mutations in key genes indeed differ according to morphology and that emphasis on morphology rather than anatomy is of importance. Of particular potential clinical relevance are the findings that assessment of *KRAS* mutation may add value to prognostication of patients with intestinal type tumors and that *SMARCA4/*BRG1 expression may be of use as a predictive biomarker for patients with pancreatobiliary-type tumors receiving chemotherapy.
- Thus, this study further underlines that molecular profiling in combination with assessment of tumor morphology may be a useful tool for improved treatment stratification of patients with adenocarcinoma in the periampullary region.

### Ethics Approval and Consent to Participate

All national and European Union regulations and requirements for handling human samples have been fully complied with during the conduct of this study, ie, decision number 1110/94/EC of the European Parliament and of the Council (OJL126 18,5,94), the Helsinki Declaration on ethical principles for medical research involving human subjects, and the European Union Council Convention on human rights and biomedicine. Approval for the study was obtained from the ethics committee of Lund University (reference number 445/07), whereby the committee waived the need for consent other than the option to opt ;out.

### Next-Generation Sequencing

Tissue cores of 1-mm diameter were taken from tumor cell–enriched formalin-fixed paraffin-embedded tissue. DNA extraction was performed using the Qiagen GeneRead (Qiagen, Hilden, Germany) kit for formalin-fixed paraffin-embedded tissue according to the manufacturer’s instructions. In total, 102 (58.3%) cases had a sufficient number of tumor cells for analysis. The anatomic site of origin of these tumors was 10 duodenal, 42 ampullary, 30 distal bile duct, and 20 pancreatic. Forty-one (39.8%) were classified as I-type and 62 (60.2%) as PB-type.

A panel of 70 cancer-associated genes was put together for this study and characterized using Illumina TruSeq custom amplicon assay (Illumina, San Diego, CA) with a MiSeq instrument according to manufacturer’s instructions. The gene panel was selected to include known cancer-associated genes and is detailed in the Data Supplement. The panel was designed to be bidirectional and to target the most cancer-relevant parts of the selected genes. Only exon parts of the aforementioned genes were sequenced. Alignment, quality filtering, variant calling, and variant annotation were performed using the supplier’s standard analysis pipeline (Illumina, San Diego, CA). Only nonsynonymous variants with variant frequency of 4% or more were not included. Detected mutations were screened against the COSMIC and ExAC databases, to filter single-nucleotide polymorphisms commonly reported in different populations.

### Immunohistochemical Analysis of Brahma-Related Gene 1 Protein Expression

For immunohistochemical analysis of Brahma-related gene 1 (BRG1) protein expression, 4-μm tissue microarray sections were automatically pretreated in the PT-link system (Dako, Glostrup, Denmark) and stained in an automated immunostainer (Autostainer Plus, Dako) using the Dako EnVision FLEX+ Detection System, Peroxidase/DAB, Rabbit/Mouse with the monoclonal anti-BRG1 antibody clone G-7 (Santa Cruz Biotechnology, Dallas, TX), diluted 1:25.

BRG1 was expressed in the tumor cell nuclei and present in the majority of tumor cells in positive cases. Therefore, only the intensity was annotated as 0 = negative, 1 = weak, 2 = moderate, and 3 = strong. Each tissue microarray core was evaluated separately, and the lowest and highest scores were denoted for each case. For the statistical analyses, a total score (0 to 6) was calculated from the sum of the lowest and highest scores. On the basis of visual inspection of Kaplan-Meier curves for the entire cohort and in strata according to morphology and adjuvant treatment, the total score was dichotomized into low (0 or 1) versus high (> 1) BRG1 staining.

### Statistical Analyses, Data Processing, and Data Availability Statement

The χ^2^ test was applied to compare the distribution of clinicopathological factors in cases with information on mutational status and cases without information. Classification and regression tree analysis was applied to find an optimal prognostic cutoff for mutation burden. Kaplan-Meier analysis log-rank test was applied to illustrate any difference in 5-year overall survival (OS), and Cox regression proportional hazard models were used to estimate hazard ratios (HRs) for death within 5 years in both univariable and multivariable analysis. Multivariable Cox regression included adjustment for age (continuous), T-stage (T1 or T2 *v* T3 or T4), N-stage (negative *v* positive nodal status), grade (well to moderate *v* poor), morphology (I-type *v* PB-type) in the entire cohort, adjuvant chemotherapy (none *v* any), invasion into vascular and lymphatic structures, and perineural growth. The proportional hazard (PH) assumption was tested using Cox regression with a time-dependent covariate analysis, whereby the PH assumption was considered to be satisfied when the factor × time interaction was nonsignificant. The PH assumption was also evaluated graphically using log-minus-log plots. In the entire cohort, three cases were excluded in the survival analyses: one patient with a PB-type tumor who was lost to follow-up because of emigration, and two patients with I-type tumors who died as a result of complications from the initial surgical treatment. In the group with information on mutational status, one patient who died as a result of complications from surgery was censored. Two additional patients with PB-type tumors having received neoadjuvant chemotherapy were excluded from the survival analyses related to BRG1 expression. To estimate the interaction effect between adjuvant treatment and selected biomarkers, the following interaction variable was constructed: any adjuvant treatment (+/−) × biomarker (+/−). Genes that were mutated in less than 10% of the cases were excluded from the survival analyses to obtain reasonable statistical power. All calculations were performed with SPSS version 24.0 (SPSS, Chicago, IL). OncoPrinter at cBioPortal (http://www.cbioportal.org/) was used to illustrate the data with heat maps.^10,11^ All statistical tests were two-sided, and *P*-values < 0.05 were considered significant. All data generated or analyzed during this study are included in this published article (Data Supplement).

### 

## RESULTS

### Distribution of Mutations According to Morphologic Type

**FIG 1.**
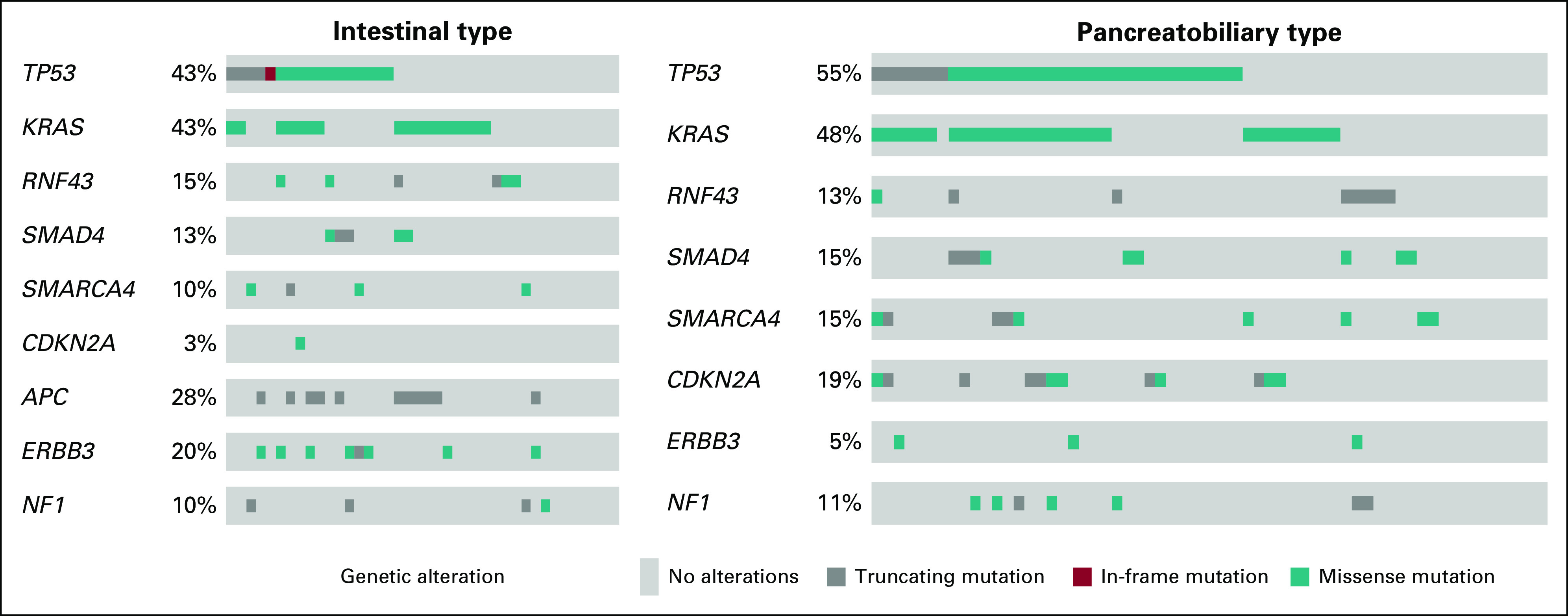
The frequency and type of mutations by morphologic type. Heat maps illustrate the frequency and type of mutations in intestinal-type and pancreatobiliary-type tumors. The genetic alterations were classified as either truncating, missense, or in-frame mutations.

There were no significant differences in the distribution of clinicopathological characteristics between cases with known and unknown mutational status (Data Supplement). The frequency of mutations according to morphologic type is shown in Figure 1 and further outlined in the Data Supplement. In the entire cohort, only nine out of 70 genes were mutated in more than 10% of the cases; the most common were *TP53* (n = 51; 50.0%) and *KRAS* (n = 47; 46.1%). *APC* mutations were significantly more common in I-type compared to PB-type tumors (27.5% *v* 0%; *P* < .001), and *CDKN2A* mutations were significantly more common in PB-type compared to I-type tumors (19.4% *v* 2.5%; *P* = .013*).* Furthermore, *ERBB3* mutations were more frequent in I-type compared with PB-type tumors (20.0% *v* 4.8%; *P* = .016). Human epidermal growth factor receptor 3 (HER3) protein overexpression has previously also been found to be significantly more frequent in I-type compared with PB-type tumors,^9^ but there was no significant association between HER3 expression and *ERBB3* mutational status (*P* = .67). The frequency of mutations according to anatomic origin is shown in the Data Supplement.

### Prognostic Impact of the Most Frequent Mutations

Kaplan-Meier analyses demonstrate a significantly prolonged OS for patients with I-type compared with patients with PB-type tumors (Data Supplement). When stratifying for anatomic origin, the survival curves cluster into two groups concordant with morphology (Data Supplement).

Of the nine most frequently mutated genes outlined above, only *APC*, *ERBB3*, *KRAS*, and *SMARCA4* were shown to confer a prognostic value, depending on the context. Both *APC* and *ERBB3* mutations were significantly associated with a prolonged OS in the entire cohort in univariable, but not in multivariable, analysis (HR, 0.30; 95% CI, 0.11 to 0.82; and HR, 0.34; 95% CI, 0.12 to 0.92, respectively). *APC* mutation was not prognostic in I-type tumors (data not shown), and *ERBB3* mutation was not prognostic in analyses stratified for morphology (data not shown).

**FIG 2.**
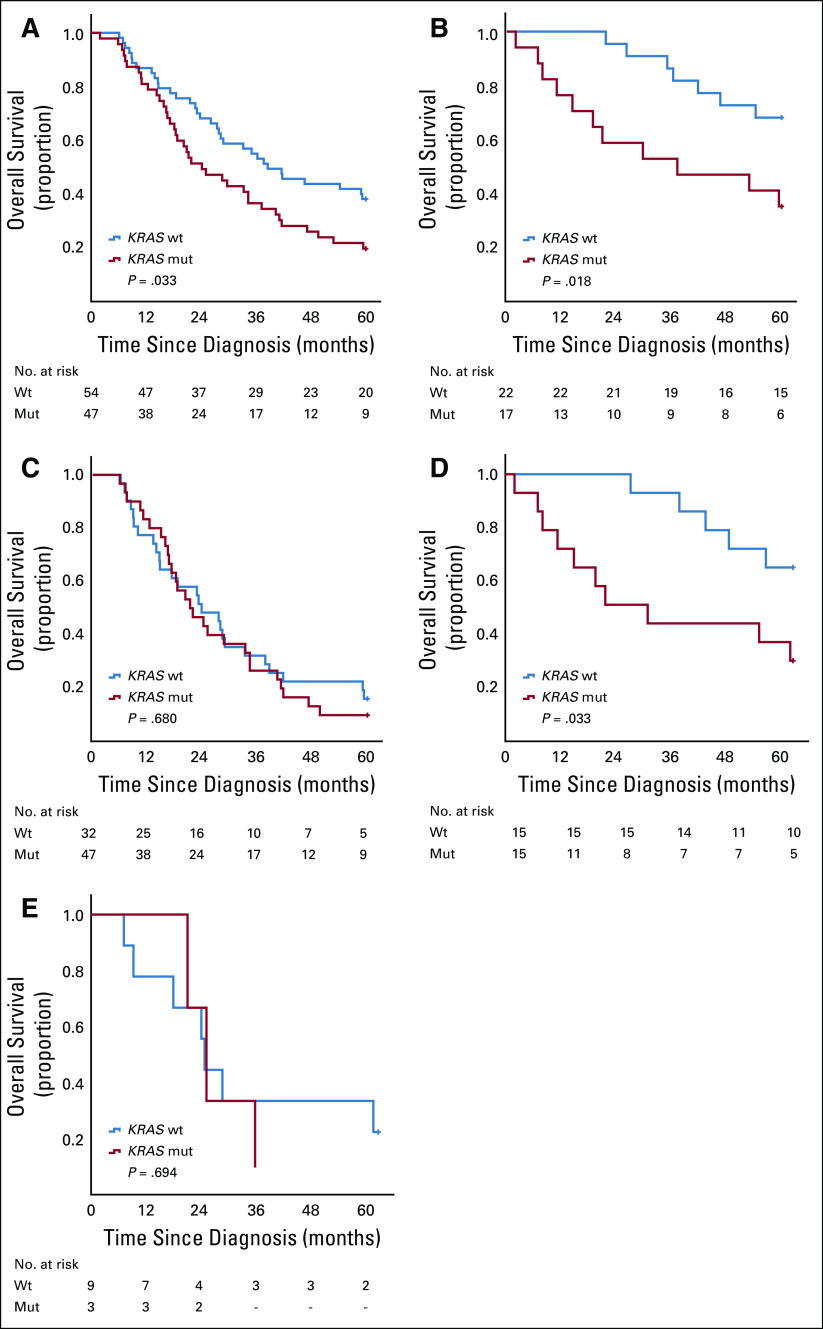
Relationship of *KRAS* mutation status with overall survival in the entire cohort and according to morphology. Kaplan-Meier curves visualize differences in 5-year overall survival for patients with *KRAS*-mutated (mut) and wild-type (wt) tumors in (A) the entire cohort, (B) intestinal-type tumors, (C) pancreatobiliary-type tumors, (D) intestinal-type ampullary tumors, and (E) pancreatobiliary-type ampullary tumors.

*KRAS* mutation was the strongest predictor of survival, as visualized in Figure 2. *KRAS* mutation was significantly associated with a reduced OS in the entire cohort (*P* = .029; Fig 2A). This association was confirmed in univariable Cox regression analysis (HR, 1.68; 95% CI, 1.05 to 2.68) and remained significant in multivariable analysis (HR, 1.67; 95% CI, 1.01 to 2.73). *KRAS* mutation was also a significant factor for reduced OS in I-type tumors (*P* = .018; Fig 2B), but not in PB-type tumors (Fig 2C). This association was confirmed in univariable Cox regression analysis (HR, 3.00; 95% CI, 1.16 to 7.52) and remained significant in multivariable analysis (HR, 3.73; 95% CI, 1.10 to 12.67). When stratifying for ampullary origin, *KRAS* mutation remained a prognostic factor in I-type tumors (*P* = 0.033; Fig 2D) but was not prognostic in PB-type tumors (Fig 2E). This association was confirmed in univariable Cox regression analysis (HR, 3.06; 95% CI, 1.04 to 9.00) and remained significant in multivariable analysis (HR, 7.76; 95% CI, 1.37 to 43.95).

**FIG 3.**
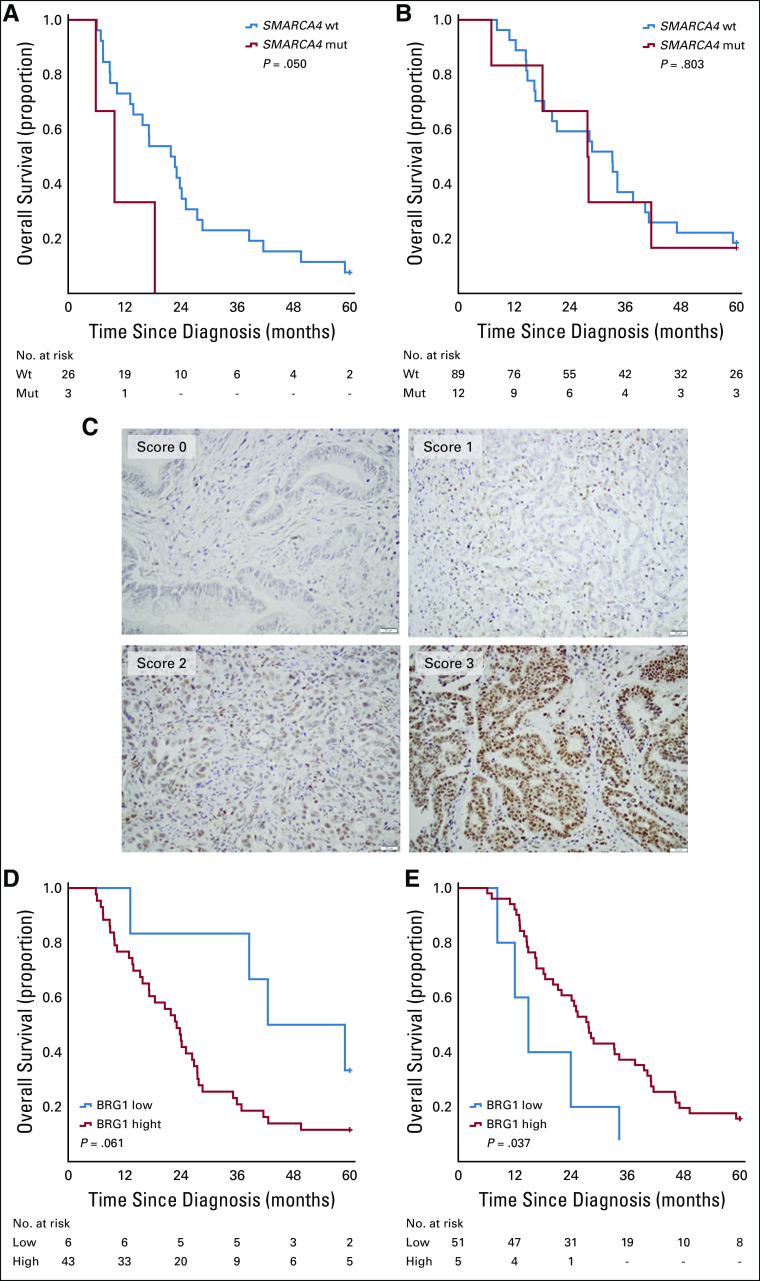
Relationship of *SMARCA4* mutation status and Brahma-related gene 1 (BRG1) protein expression with overall survival according to adjuvant chemotherapy in patients with pancreatobiliary (PB)-type tumors. (A, B) Kaplan-Meier curves visualizing differences in 5-year overall survival in patients with PB-type tumors according to *SMARCA4* mutation status (A) without adjuvant chemotherapy, and (B) with adjuvant chemotherapy. (C) Sample immunohistochemical images of BRG-1 expression. (D, E) Kaplan-Meier analyses visualizing differences in 5-year overall survival in patients with PB-type tumors according to low and high BRG1 expression (D) without adjuvant chemotherapy, and (E) with adjuvant chemotherapy. mut, mutated; wt, wild type.

*SMARCA4* mutation was not prognostic in the entire cohort or in strata according to morphology (data not shown). However, as shown in Figure 3, *SMARCA4* mutation was significantly associated with a shorter OS in patients with PB-type tumors who did not receive adjuvant chemotherapy (*P* = .050; Fig 3A), but there was no significant interaction between *SMARCA4* mutation and adjuvant treatment. *SMARCA4* mutation was not prognostic in patients with PB-type tumors who received adjuvant chemotherapy (Fig 3B), and the prognostic value of *SMARCA4* mutation did not differ according to adjuvant chemotherapy in I-type tumors (data not shown). Given the potential relationship of *SMARCA4* mutation with chemotherapy seen in PB-type tumors, we proceeded to examine the prognostic value of immunohistochemical expression of BRG1, the protein encoded by the *SMARCA4* gene, in tissue microarrays with samples from primary tumors (n = 175) from the original cohort.^9^ In all, BRG1 expression could be evaluated in 170 (97.1%) cases. Sample immunohistochemical images are shown in Figure 3C. In the entire cohort, high BRG1 expression was an adverse prognostic factor in patients not receiving adjuvant chemotherapy but was not prognostic in patients receiving adjuvant chemotherapy (data not shown). Subgroup analysis revealed that BRG1 was only prognostic in PB-type tumors, depending on adjuvant chemotherapy (Figs 3D and 3E), and there was a significant interaction between BRG1 expression (0 or 1 *v* > 1) and adjuvant treatment in PB-type tumors (*P* for interaction = .007). BRG1 was not prognostic in I-type tumors overall or in strata according to chemotherapy (data not shown). BRG1 expression was significantly higher in PB-type, but not in I-type, tumors harboring *SMARCA4* mutations compared with wild type. The mutational burden of the analyzed genes did not differ significantly between I-type and PB-type tumors (*P* = .577) and was not prognostic in the whole cohort or stratified for morphologic type.

## DISCUSSION

Morphology is emerging as an important prognostic factor in periampullary adenocarcinoma,^1,2^ and to our knowledge, this is the first study to comprehensively map common cancer-associated mutations in the full range of periampullary adenocarcinoma, with particular reference to morphology. Herein, we describe that the pattern of the mutational status, such as *CDKN2A* and *APC* mutations, of periampullary adenocarcinomas differs by morphologic subtype. These findings are in line with a study on 112 cancers of ampullary origin, demonstrating that the mutational spectrum in I-type tumors resembles that of colorectal cancer, and the mutational spectrum in PB-type tumors resembles that of pancreatic cancer.^12^ In the current study, mutations in *APC* but not in *CDKN2A* were found to be prognostic. Given that no PB-type tumors harbored *APC* mutations, it is plausible to assume that the link between these mutations and a favorable outcome in the entire cohort is mainly due to their association with I-type morphology. The same line of reasoning applies to the association between *ERBB3* mutations, being more prevalent in I-type tumors, and a prolonged survival in the entire cohort. Adding to this, there was no significant association between HER3 expression and *ERBB3* mutational status, and the clinical utility of assessment of *ERBB3* mutation status for prognostication purposes needs additional validation.

*KRAS* mutation was found to signify a significantly shorter survival in patients with I-type tumors, also when adjusted for established clinical factors, whereas patients with *KRAS* wild-type I-type tumors could indeed be classified as long-term survivors. Two other studies have demonstrated *KRAS* mutations to be associated with poor prognosis in ampullary cancer;^13,14^ however, none of these considered tumor morphology. As of yet, epidermal growth factor receptor (EGFR) inhibition has not shown clinical efficacy in pancreatic cancer,^15^ but no study has investigated the efficacy of EGFR inhibitors in periampullary adenocarcinoma in relation to morphology. Although none of the patients in this study had received EGFR-inhibiting treatment, the findings indicate that some *RAS* wild-type periampullary cancers of I-type may indeed benefit from such treatment. Furthermore, the findings highlight a pressing need to identify optimized and individualized treatment protocols for patients with these tumors according to morphology and mutational status and that molecular profiling of these tumors may be of clinical benefit.

The only gene mutation for which the prognostic significance was found to differ by adjuvant treatment was *SMARCA4*, where a significant association was observed between *SMARCA4* mutation and reduced survival in patients with PB-type tumors not having received adjuvant treatment. Despite the lack of a significant interaction between *SMARCA4* mutation and adjuvant treatment, this observation led us to further explore the expression and prognostic value of BRG1, the protein encoded by *SMARCA4*, in tumors from the full study cohort. BRG1 was found to be an adverse factor in patients who did not receive adjuvant chemotherapy and, in addition, there was a significant interaction with adjuvant chemotherapy in PB-type tumors. BRG1 is an ATP-dependent chromatin remodeling protein^16^ that has been sparsely studied in periampullary cancer. One study on pancreatic cancer (n = 68) failed to demonstrate an association between BRG1 expression and gemcitabine response or survival,^17^ but the finding of a potential predictive role for *SMARCA4/*BRG1 in the current study merits further validation in additional patient cohorts.

To our knowledge, this is the first study to map the prevalence and prognostic significance of mutations in common cancer-associated genes in the full spectrum of periampullary adenocarcinoma. The findings demonstrate that the distribution and prognostic significance of some of the most commonly mutated genes differ by morphologic type. In particular, *KRAS* mutation status may be a suitable biomarker for prognostication in patients with I-type tumors, and *SMARCA4*/BRG1 expression may add value in the prediction of response to adjuvant chemotherapy treatment in patients with PB-type tumors. Moreover, the study supports that the use of molecular profiling could be of clinical benefit for patients with these types of tumors.

## References

[1] Westgaard A Tafjord S Farstad IN et al Pancreatobiliary versus intestinal histologic type of differentiation is an independent prognostic factor in resected periampullary adenocarcinoma BMC Cancer 8 170 2008 18547417 10.1186/1471-2407-8-170 PMC2430209

[2] Bronsert P Kohler I Werner M et al Intestinal-type of differentiation predicts favourable overall survival: Confirmatory clinicopathological analysis of 198 periampullary adenocarcinomas of pancreatic, biliary, ampullary and duodenal origin BMC Cancer 13 428 2013 24053229 10.1186/1471-2407-13-428 PMC3849372

[3] Kimura W Futakawa N Zhao B Neoplastic diseases of the papilla of Vater J Hepatobiliary Pancreat Surg 11 223 231 2004 15368105 10.1007/s00534-004-0894-7

[4] Waddell N Pajic M Patch AM et al Whole genomes redefine the mutational landscape of pancreatic cancer Nature 518 495 501 2015 25719666 10.1038/nature14169 PMC4523082

[5] Bailey P Chang DK Nones K et al Genomic analyses identify molecular subtypes of pancreatic cancer Nature 531 47 52 2016 26909576 10.1038/nature16965

[6] Moffitt RA Marayati R Flate EL et al Virtual microdissection identifies distinct tumor- and stroma-specific subtypes of pancreatic ductal adenocarcinoma Nat Genet 47 1168 1178 2015 26343385 10.1038/ng.3398 PMC4912058

[7] Collisson EA Sadanandam A Olson P et al Subtypes of pancreatic ductal adenocarcinoma and their differing responses to therapy Nat Med 17 500 503 2011 21460848 10.1038/nm.2344 PMC3755490

[8] Elebro J Jirström K Use of a standardized diagnostic approach improves the prognostic information of histopathologic factors in pancreatic and periampullary adenocarcinoma Diagn Pathol 9 80 2014 24731283 10.1186/1746-1596-9-80 PMC3999361

[9] Elebro J Heby M Warfvinge CF et al Expression and prognostic significance of human epidermal growth factor receptors 1, 2 and 3 in periampullary adenocarcinoma PLoS One 11 e0153533 2016 27070783 10.1371/journal.pone.0153533 PMC4829175

[10] Gao J Aksoy BA Dogrusoz U et al Integrative analysis of complex cancer genomics and clinical profiles using the cBioPortal Sci Signal 6 pl1 2013 23550210 10.1126/scisignal.2004088 PMC4160307

[11] Cerami E Gao J Dogrusoz U et al The cBio cancer genomics portal: An open platform for exploring multidimensional cancer genomics data Cancer Discov 2 401 404 2012 22588877 10.1158/2159-8290.CD-12-0095 PMC3956037

[12] Yachida S Wood LD Suzuki M et al Genomic sequencing identifies ELF3 as a driver of ampullary carcinoma Cancer Cell 29 229 240 2016 26806338 10.1016/j.ccell.2015.12.012 PMC5503303

[13] Schultz NA Roslind A Christensen IJ et al Frequencies and prognostic role of KRAS and BRAF mutations in patients with localized pancreatic and ampullary adenocarcinomas Pancreas 41 759 766 2012 22699145 10.1097/MPA.0b013e31823cd9df

[14] Valsangkar NP Ingkakul T Correa-Gallego C et al Survival in ampullary cancer: Potential role of different KRAS mutations Surgery 157 260 268 2015 25616942 10.1016/j.surg.2014.08.092 PMC4451831

[15] Philip PA Benedetti J Corless CL et al Phase III study comparing gemcitabine plus cetuximab versus gemcitabine in patients with advanced pancreatic adenocarcinoma: Southwest Oncology Group-directed intergroup trial S0205 J Clin Oncol 28 3605 3610 2010 20606093 10.1200/JCO.2009.25.7550 PMC2917315

[16] Narlikar GJ Fan HY Kingston RE Cooperation between complexes that regulate chromatin structure and transcription Cell 108 475 487 2002 11909519 10.1016/s0092-8674(02)00654-2

[17] Numata M Morinaga S Watanabe T et al The clinical significance of SWI/SNF complex in pancreatic cancer Int J Oncol 42 403 410 2013 23229642 10.3892/ijo.2012.1723 PMC3583622

